# Editorial: Advances in artificial intelligence-enhanced electrocardiography: a pathway towards improved diagnosis and patient care

**DOI:** 10.3389/fphys.2025.1687592

**Published:** 2025-09-05

**Authors:** Vijay S. Chauhan, Rémi Dubois, Domenico L. Gatti, Jichao Zhao

**Affiliations:** ^1^ Peter Munk Cardiac Centre, University Health Network (UHN), Toronto, ON, Canada; ^2^ IHU LIRYC: Cardiac Electrophysiology and Heart Modeling Institute, Bordeaux, France; ^3^ School of Medicine, Wayne State University, Detroit, MI, United States; ^4^ The University of Auckland, Auckland, New Zealand

**Keywords:** ECG, electrocardiography, machine learning (ML), artificial intelligence, signal processing (SP)

## Introduction

In the unfolding landscape of digital health, artificial intelligence (AI) is rapidly redefining the ways in which biomedical data is interpreted and utilized for clinical decision-making. Among the myriad biomedical signals analyzed using AI, the electrocardiogram (ECG) has emerged as a particularly fertile area of investigation (Wu and Guo, 2025; Oke and Cavus, 2025). Its noninvasive nature, ubiquity, and relatively standardized data format make it ideal for machine learning (ML) and deep learning (DL) applications. Over the past decade, AI-enhanced ECG interpretation has progressed from a research endeavor to a clinical reality. Early models primarily demonstrated classification capabilities under tightly controlled conditions. Today, the research community is grappling with more complex but necessary challenges: model generalizability across populations and devices, regulatory pathways, integration into clinical workflows, and ethical issues around trust, bias, and accountability (Silva et al., 2025).

The Research Topic “Advances in Artificial Intelligence Enhanced Electrocardiography” was conceived to provide a forum for the latest innovations in this rapidly advancing field. Our call for submissions focused on a wide array of themes, including model development, signal preprocessing, real-time application, interpretability, and translational research.

The articles collected in this Research Topic reflect the richness and depth of ongoing work at this critical intersection of cardiology, computer science, and biomedical engineering. They span applications ranging from real-time arrhythmia detection in portable devices to noninvasive fetal heart monitoring, from advanced signal preprocessing to optimized neural network input strategies. While each article offers a distinct perspective, collectively, the articles in this Research Topic highlight how AI is reshaping the acquisition, interpretation, and deployment of ECG-based diagnostics.

In a major step toward practical deployment, Panwar et al. developed an end-to-end portable ECG monitoring system capable of classifying arrhythmias in real-time using a convolutional neural network (CNN). What distinguishes this work is its integration with a microcontroller-based hardware platform (Arduino), demonstrating that AI-based cardiac monitoring need not rely on cloud computing or high-end hardware, and positioning the system as a potential solution for rural health monitoring or home-based cardiac care.

Ruppersberg et al. address a pressing challenge in electrophysiology: identifying non-pulmonary vein (non-PV) drivers of persistent atrial fibrillation (AF). This study employs machine learning-enhanced electrographic flow (EGF) mapping from over 400 patient cases, integrating optical-flow physics with data-driven classification, to establish a clinically relevant threshold for source localization. This work demonstrates how AI can refine mechanistic understanding of complex arrhythmias and assist clinicians in tailoring ablation strategies.

Electrode positioning accuracy is foundational to high-resolution ECG mapping techniques such as Body Surface Potential Mapping (BSPM). The technical requirements of BSPM have traditionally limited its clinical adoption. El Ghebouli et al. confront this challenge by proposing a camera-based approach to ECG electrode localization. Using 2D and 3D computer vision algorithms, the authors demonstrate sub-centimeter accuracy in reconstructing electrode positions on the human torso. The study’s reliance on off-the-shelf cameras and open-source algorithms makes it a scalable solution for BSPM expansion.

Kim et al. focus on scanned or imaged ECG paper strips, which are still widely used in many healthcare settings. Their two-stage deep learning system combines a Faster R-CNN for detecting ST-segment elevation with an ensemble model for infarction territory classification. This paper underscores a growing priority in AI research: meeting clinicians where they are. By enabling analysis of ECG images (rather than raw digital signals), the model is inherently compatible with a wide array of existing workflows, including those in resource-constrained environments, and is particularly attractive for frontline decision support.

Neural network performance depends critically on the quality and relevance of input data. Ramirez et al. explore this issue by applying a mutual information analysis to ECG leads, identifying redundant information across the 12 standard leads and testing various reduced-lead configurations. Their results reveal that a well-selected 6-lead subset, and vectorcardiographic transformations, can match or exceed full 12-lead performance, and preserve classification accuracy while reducing computational load. This has significant implications for wearable devices and mobile health, where data acquisition may be limited. By optimizing for both informativeness and parsimony, this work advances the efficiency and scalability of AI-driven ECG diagnostics.

QRS detection is a fundamental building block in any ECG analysis pipeline. Zhao et al. contribute a compact, yet highly accurate DNN model based on feature pyramid networks and dual-channel input. The model’s minimal size (∼27k parameters) and fast inference make it ideal for edge computing applications, such as smartwatches, fitness trackers, or implantable devices. This work reinforces the emerging consensus that the future of AI-enhanced ECG lies in small, explainable, and highly optimized models tailored to specific tasks within broader clinical systems.

Extending the reach of AI-enhanced ECG into maternal-fetal medicine, Wahbah et al. present a bi-directional LSTM-based framework for extracting fetal ECG (fECG) signals from abdominal recordings. Their model achieves high accuracy and demonstrates resilience even during stages where the fetal signal is physiologically obscured. As fetal and neonatal ECGs pose unique signal processing challenges, this study opens new avenues for AI-assisted perinatal care, remote monitoring, and early detection of congenital abnormalities.

## Looking forward: a field poised for impact

The contributions to this Research Topic highlight a discipline on the cusp of transformation. From novel signal processing and intelligent hardware to regulatory-aware, interpretable algorithms, the field of AI-enhanced ECG analysis is advancing rapidly toward real-world impact.

However, critical challenges remain. Generalizability across diverse populations, integration with electronic health records (EHRs), and validation in prospective trials are essential next steps. Ethical considerations, especially around algorithmic bias, data privacy, and clinical accountability, must be integrated into development from the outset.

We are beginning to see new frontiers: multimodal integration (combining ECG with imaging, labs, or genomics), personalized risk prediction, and AI-guided therapeutic interventions. As Editors of this Research Topic, we are inspired by the diversity, creativity, and clinical awareness shown by the authors in this issue. The articles not only advance the science of AI in ECG analysis but also illuminate the path to meaningful clinical translation. Together, their work illustrates a maturing ecosystem of tools, methods, and philosophies ready to shape the next era of cardiovascular care.
